# Development, Survival, and Reproduction of *Orius insidiosus* (Hemiptera: Anthocoridae) Reared on *Frankliniella invasor* (Thysanoptera: Thripidae)

**DOI:** 10.1093/jisesa/ieab030

**Published:** 2021-05-11

**Authors:** Lucia Carrillo-Arámbula, Francisco Infante

**Affiliations:** El Colegio de la Frontera Sur (ECOSUR), Carretera Antiguo Aeropuerto km 2.5, Tapachula, 30700 Chiapas, México

**Keywords:** biocontrol, predation, Anthocoridae, Thripidae

## Abstract

The minute pirate bug *Orius insidiosus* (Say) is an important predator in mango agroecosystems. It attacks several species of thrips, particularly *Frankliniella invasor* Sakimura, which is considered a species of economic importance in mango. We investigated the effect of six diets on the development, survival, and reproduction of *O. insidiosus*: 1) first instars of *F. invasor*, 2) second instars, 3) adults, 4) pollen, 5) pollen plus thrips larvae, and 6) water. Individuals fed on thrips larvae, with or without pollen, completed their immature development significantly faster. Nymphs of *O. insidiosus* were able to complete their development feeding on pollen only, while individuals that received water as a diet were unable to reach the adult stage. The highest intrinsic growth rate was obtained when *O. insidiosus* were fed on pollen plus thrips larvae, and the lowest when individuals were fed on thrips adults. Our studies show that a diet of pollen plus *F. invasor* larvae is optimal for *O. insidiosus* development and population growth.

*Frankliniella invasor* Sakimura (Thysanoptera: Thripidae) is a phytophagous thrips that can be found in high densities on mango panicles in several countries of Central and South America (Rocha et al. 2012). This species feeds and reproduces in flowers. Mango panicles under a severe attack of thrips quickly dry and drop flowers, affecting fruit set and yields. Population outbreaks of *F. invasor* are typically managed by insecticide sprays, but alternative methods of control are needed. Rocha et al. (2015) listed the natural enemies of *F. invasor,* and noted that the minute pirate bug, *Orius insidiosus* (Say) (Hemiptera: Anthocoridae), was among its most important predators. To develop a sustainable crop protection strategy against thrips in mango, it is necessary to enhance the use of beneficial insects by means of augmentative biological control. This typically involves the rearing and release of natural enemies to decrease pest populations. Several rearing systems for *O. insidiosus* have been proposed using different types of prey (Tommasini et al. 2004, Bueno et al. 2006). However, rearing natural enemies on prey or hosts that are different to the target pest may lead to changes in their physiology or behavior, and therefore reduce their efficiency to control the pest (Grenier and De Clercq 2003). This study investigated the suitability of *F. invasor* for rearing *O. insidiosus* as a biological control agent. The purpose was to evaluate the effect of different biological stages of *F. invasor* on the reproductive parameters of *O. insidiosus* under laboratory conditions.

## Materials and Methods

Adults of *O. insidiosus* were gathered from blooming cornfields (*Zea mays* L.) near mango plantations on February 2018. Specimens were caught at Ejido el Manzano in Chiapas, México. Corn spikes were shaken against a plastic tray to separate insects, and pirate bugs were collected and placed individually in vials. Once in the laboratory, individuals of *O. insidiosus* were separated from other anthocorids following the taxonomic keys by Herring (1966) and Shapiro et al. (2010). A group of 75 *O. insidiosus* adults (males and females) was placed in a 26 × 14 × 12 cm ventilated container, and corrugated paper was added to reduce cannibalism. Water (wet cotton), pollen, and dipteran eggs were provided ad libitum as food and replaced every other day. The pollen used was a dehydrated mixture of many tropical plants (Abel-Ha; Naucalpan, México), and the eggs were of *Anastrepha ludens* Loew (Diptera: Tephritidae), which were provided by MOSCAFRUT mass-rearing facilities located in Metapa, Chiapas. Fresh snap bean pods, *Phaseolus vulgaris* L., were used as oviposition substrate. The snap beans were replaced every 12 h, and *O. insidiosus* eggs were counted under a stereomicroscope.

*Orius insidiosus* eggs used for experiments were taken from the reproduction containers described above, and *F. invasor* was collected from mango orchards. The developmental time and survival of *O. insidiosus* were evaluated on six food treatments: 1) first instars of *F. invasor*, 2) second instars, 3) adults, 4) pollen, 5) pollen + thrips larvae, and 6) water. The experiment was initiated with 50 individuals per treatment. After eclosion, newborn *O. insidiosus* nymphs (<12 h) were placed individually in plastic cups (6 cm diam) and assigned at random to one of the six treatments. The number of prey larvae offered was adjusted gradually, first and second instars of *O. insidiosus* were offered five larvae per experimental unit. Third and fourth instars were offered 10 larvae, and the fifth instars and adults were offered 15 larvae. Development and survival of *O. insidiosus* were recorded daily from eclosion to adult emergence. Experiments were conducted at 29 ± 5°C, 50–60% RH, and a photoperiod of 14L:10D. The mortality rates of *O. insidiosus* were compared through a χ ^2^ test for homogeneous proportions and the Kruskal–Wallis test was applied to compare differences in the development time.

The effect of diet on the reproduction and longevity of adult females was assessed with a variable number of individuals, as we used the adults that emerged from each particular treatment in the previous experiment. We had the following treatments and number of females: 1) first instars of *F. invasor* (*n* = 11), 2) second instars (*n* = 11), 3) adults (*n* = 7), 4) pollen (*n* = 15), and 5) pollen plus thrips larvae (*n* = 10). Females were placed individually inside plastic cups with a snap bean as an oviposition substrate, and one male was added to ensure female fertility. The diet and snap beans were replaced daily. Demographic parameters were calculated from daily records of mortality and progeny production of *O. insidiosus*, and data were summarized in a life fertility table (Southwood 1966, Carey 1993). Survival data were analyzed through the exact and asymptotic weighted log-rank test for interval censored data using the R statistical package.

## Results

The type of diet significantly influenced the development of *O. insidiosus* (Table 1). Individuals that were fed on thrips larvae, or on thrips larvae plus pollen, completed their development significantly faster than other treatments (χ ^2^ = 97.45; df = 4; *P* < 0.001). Nymphs of *O. insidiosus* were able to complete development feeding on pollen only. The longest development was recorded when individuals fed on pollen, and the shortest when fed on second instars of *F. invasor*. Individuals that received water as a diet were unable to reach the adult stage. The type of diet had a significant influence on the mortality (χ ^2^ = 88.92; df = 5; *P* < 0.001). Nymphs fed on pollen plus thrips larvae had the lowest overall mortality (22%), while individuals that were maintained without food (water) had 100% mortality.

**Table 1.** Developmental time (days) of the minute pirate bug *Orius insidiosus* fed on six diets

Instar	Diet					
	Water	Pollen	L_1_	L_2_	Adult	Pollen + L_1_ + L_2_
I	2.54 ± 1.06c	2.76 ± 0.11cd	1.17 ± 0.08ab	1.15 ± 0.07a	2.56 ± 0.17cd	1.36 ± 0.09ab
	*n* = 24	*n* = 47	*n* = 42	*n* = 48	*n* = 39	*n* = 50
II	9 ± 1.41d	5.09 ± 0.27c	2.30 ± 0.08ab	2.06 ± 0.07ab	2.27 ± 0.18ab	1.92 ± 0.07a
	*n* = 2	*n* = 42	*n* = 40	*n* = 47	*n* = 33	*n* = 49
III	†	3.13 ± 0.25bcd	2.37 ± 0.19ab	1.83 ± 0.12a	3.71 ± 0.22d	3.29 ± 0.11cd
		*n* = 38	*n* = 38	*n* = 41	*n* = 28	*n* = 48
IV	†	2.32 ± 0.14abc	2.19 ± 0.13ab	2.67 ± 0.13c	2.48 ± 0.22abc	2.14 ± 0.09a
		*n* = 34	*n* = 37	*n* = 36	*n* = 23	*n* = 43
V	†	2.03 ± 0.08ab	2.67 ± 0.18b	2.19 ± 0.11ab	2.50 ± 0.27ab	1.79 ± 0.14a
		*n* = 34	*n* = 36	*n* = 32	*n* = 18	*n* = 39
Total	†	15.65 ± 0.26d	10.75 ± 0.3ab	9.81 ± 0.24a	13 ± 0.43c	10.46 ± 0.21ab
		*n* = 34	*n* = 36	*n* = 32	*n* = 18	*n* = 39

L_1_ = first instar, L_2_ = second instar, and adult = adult stage of *Frankliniella invasor*. Means (± SD) followed by different letters within the same row are significantly different (Tukey, *P* < 0.05).

† Individuals did not complete development as all of them died.

**Fig. 1.**
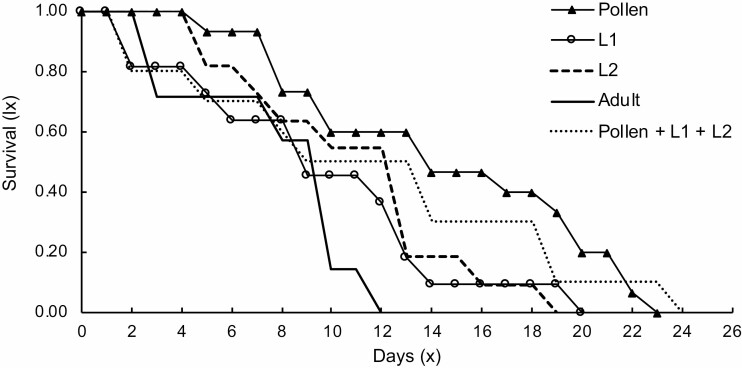
Survival curves of *Orius insidiosus* adult females fed on five diets. Median survival times (50%) did not differ among groups (log-rank test: *P* = 0.081).

Diets did not have a significant effect on the longevity of females (log-rank test; χ ^2^ = 8.27; df =4; *P* = 0.081). The median survival was reached at 13, 8, 12, 9, and 13 d, when individuals were fed on pollen, first instars, second instars, adults, and pollen + thrips larvae, respectively (Fig. 1). Among all diets, *O. insidiosus* had the best population growth when fed on pollen plus thrips larvae (Table 2).

**Table 2.** Effect of five different diets on the reproductive performance of the minute pirate bug *Orius insidiosus*

Diet	R_0_	TG	*r_m_*	λ	DT
Pollen	2.347	26.257	0.033	1.034	21.024
L_1_	1.851	18	0.035	1.035	19.987
L_2_	2.438	18.011	0.050	1.051	13.880
Adult	0.515	17.837	−0.037	0.964	−18.673
Pollen + L_1_ + L_2_	5.337	19.915	0.088	1.092	7.841

L_1_ = first instar, L_2_ = second instar, and adult = adult stage of *Frankliniella invasor*. R_0_ = net reproductive rate (*R*_*0*_ = *Σl*_*x*_*m*_*x*_); TG = generation time (*TG* = ln *R*_*0*_/*r*_*m*_); *r*_*m*_ = intrinsic growth rate (*Σl*_*x*_*m*_*x*_ exp (-*r*_*m*_)=1; λ = finite capacity for increase (λ = ℮ ^*rm*^); DT = doubling time (*DT* = In 2/*r*_*m*_).

## Discussion

Demographic parameters have been widely used to evaluate the performance of natural enemies of agricultural pests (Bellows et al. 1992). There are several studies on the population parameters of *O. insidiosus* feeding on different types of prey (Kiman and Yeargan 1985, Tommasini et al. 2004, Calixto et al. 2013). However, this is the first study on the development rates, survival, and fecundity of *O. insidiosus* using *F. invasor*. Although nymphs of *O. insidiosus* could feed on all stages of *F. invasor*, these stages were not equally suitable for the predator’s development. In fact, *F. invasor* adults were the least suitable diet for nymphal development, while thrips larvae and thrips larvae plus pollen were the most suitable diets, as they resulted in faster juvenile development of the predator.

High *O. insidiosus* mortality in the treatment containing thrips adults was explained by starvation. We noted that thrips adults moved fast inside the plastic cups, and numerous *O. insidiosus* nymphs were unable to subdue and feed on prey larger than themselves. One of the most important contributions of this work was to verify that young nymphs of *O. insidiosus* were able to live without food and with only water for more than 1 wk. Similarly, some nymphs that were fed on a pollen-only diet successfully completed their development to the adult stage. These two aspects have important implications because *O. insidiosus* could persist in agroecosystems with low prey density or even when prey is temporarily absent. For many predators, pollen feeding allows individuals to survive when prey is scarce (Van Rijn et al. 2002).

Our results on *O. insidiosus* reproduction are similar to those obtained by Shapiro and Ferkovich (2006) who reported a lifetime fecundity of 5.9 eggs per female. Other reported lifetime fecundities have been variable, depending on the prey used: 20.3 eggs/female when fed on *Sericothrips variabilis* (Beach) (Kiman and Yeargan 1985), 65.7 eggs/female with *F. occidentalis* adults (Tommasini et al. 2004), and 38.2 eggs/female with *F. occidentalis* plus pollen (Calixto et al. 2013). The effect of diets on *O. insidiosus* was particularly evident for their demographic parameters. The net reproductive rate was higher when individuals were fed on pollen plus thrips larvae, while the lowest was obtained when fed with thrips adults. In parallel, the intrinsic growth rate (*r*_m_) was 0.088 and −0.037 for the pollen plus thrips larvae and thrips adults diets, respectively. Interestingly, the diet of thrips adults was the only treatment with a negative *r*_m_ value, indicating that this diet alone is not suitable for *O. insidiosus*. In conclusion, the diet with pollen plus thrips larvae shows to be the best option to rear *O. insidiosus* under laboratory conditions. Anthocorids are among the most important predators of agricultural pests, and the use of pollen plus thrips larvae to rear *O. insidiosus* for augmentative releases may prove valuable for biological control of *F. invasor* in mango agroecosystems.
